# In-Season Consumption of Locally Produced Tomatoes Decreases Cardiovascular Risk Indices

**DOI:** 10.3390/nu15010043

**Published:** 2022-12-22

**Authors:** Ma. Josefina Ruiz de Azua, Álvaro Cruz-Carrión, Begoña Muguerza, Gerard Aragonès, Anna Arola-Arnal, María Paz Romero, Francisca Isabel Bravo, Manuel Suarez

**Affiliations:** 1 Nutrigenomics Research Group, Departament de Bioquímica i Biotecnologia, Universitat Rovira i Virgili, 43007 Tarragona, Spain; 2 Antioxidants Research Group, Food Technology Department, Agrotecnio AGROTECNIO-CERCA Center, University of Lleida, Av/Alcalde Rovira Roure 191, 25198 Lleida, Spain

**Keywords:** cardiovascular risk, lycopene, phenolic compounds, season, tomato, xenohormesis

## Abstract

Tomatoes are widely consumed worldwide at any time of the year. However, depending on the variety, they have a characteristic season. We evaluated the consequences metabolic of consumption of Ekstasis tomatoes from different geographical origin and in different seasons in Fischer 344 rats. The hepatic gene expression of key enzymes in lipid metabolism was also evaluated. Animals were classified in three photoperiods (L6, L12, and L18) and in three treatments (vehicle: VH; local tomato: LT; and non-local tomato: nLT). We measured serum metabolic parameters and the gene expression of liver enzymes related to lipid metabolism (*Acc1*, *Cpt1a*, *Had*, *Fas1*, *Srebp-1c*, *Fatp5*, *Cd36*). LT consumption in season decreased cardiovascular risk 1 and coefficient atherogenic by 1.81 (*p* = 0.031) and in L6 decreased TAG and glucose (*p* = 0.046; *p* = 0.024). The L18-LT animals had decreased total cholesterol (*p* = 0.029) and gene expression of Srebp1-c (*p* = 0.022) but increased expression of *Acc1* (*p* = 0.032). The treatments significantly affected the expression of *Acc1* and *Fas1* in the liver and the levels of serum TAG and glucose. A significant effect of photoperiod on serum concentration of glucose, insulin, HOMA index, and on the hepatic expression of *Srep1-c*, *Fas1*, and *Acc1* was observed.

## 1. Introduction

The synthesis of phytochemicals by plants is a consequence of exposure to environmental and other external stressors intended to provide a protective and adaptive response. It is known that the content of these compounds, including polyphenols, depends on the external conditions to which plants are exposed in their development and harvest. In this framework, the xenohormesis theory proposes that the heterotrophs that consume plants could detect these molecules, which would provide environmental adaptive signals, regulating pathways that provide resistance to stress. Therefore, the consumption of local and seasonal plant products would produce metabolic benefits associated with the use of phytochemicals as molecular signals for the forecast of adverse conditions, such as the adaptive modulation of enzymes and receptors of stress-response pathways [1]. 

The Mediterranean diet is a characteristic food pattern that is associated with protective effects against various chronic noncommunicable diseases, including metabolic, cardiovascular, and neurodegenerative diseases. Possibly, the beneficial effects of its adherence are associated with the large amount of bioactive compounds present, their actions in the cell cycle, apoptosis and angiogenesis, and their protective properties against oxidative stress, inflammation, and metastasis [2,3,4,5,6].

The tomato is one of the vegetables most consumed within this diet. In 2020, approximately 22 million tons of tomatoes were produced throughout Europe, of which 4 million tons were of Spanish origin [7]. Interestingly, the tomato is a source of significant amounts of phytochemicals, among which are carotenoids (lycopene, phytoene, and α and β-carotene) and polyphenols (flavonoids, flavones, flavonones, and anthocyanidins) mainly [8], which are associated with several positive effects against cardiovascular diseases [9]. The amount and type of bioactive compounds in tomatoes is influenced either by genetic and agricultural and environmental conditions during growth and development that generate differences when comparing different species as well as when comparing the same type of fruit but of different geographical origin [10]. In this sense, it has been observed that the lycopene content varied from 1 to 11 mg/100 g while the amount of phenolic compounds varied between 9 to 27 mg/100 g and 25 to 50 mg/100 g in tomatoes from India or Spain, respectively [11,12]. Likewise, a 1- to 4-fold variation in ascorbic acid levels was documented between tomatoes of the same species [12].

In the last decade, the fundamental role of circadian and circannual rhythms in the regulation of physiological and metabolic processes has been claimed. In this sense, chrononutrition studies the interaction of biological rhythms, nutrients, and metabolism, focusing on how the biological clock can influence the signaling and modulation of nutrients on the body and, in turn, how nutrients and biocompounds present in food can affect our biological clock in the same way [13]. In this regard, the impact of chrononutrition on energy expenditure, thermogenesis, satiety, and the levels of serum parameters linked to the appearance and/or prevention of chronic metabolic diseases has been evidenced [14]. In our previous studies, a differential effect of the consumption of fruits in and out of season was observed. Specifically, the consumption of cherries and tomatoes in-season could prevent oxidative stress by improving antioxidant status, decreasing serum transaminase activity, and maintaining a constant level of reactive oxygen species and serum malondialdehyde [15,16]. Furthermore, the intake of orange out-of-season increased lipogenesis and decreased β-oxidation compared to its consumption in-season [17].

Therefore, considering the xenohormesis theory that fruits contain phytochemicals that would function as adaptive signals from the environment, in the present study, we aim to evaluate the effects on biochemical parameters and on the gene expression of liver enzymes of the tomato intake from different areas of Spain and its consumption in different photoperiods, which would simulate the days of the different seasons. Of note, that the use of the term in season refers to the characteristic photoperiod of the tomato harvest and consumption.

## 2. Materials and Methods

### 2.1. Fruit Material

In the present study, tomatoes fruits (*Solanum lycopersicum* cv. *Ekstasis*) grown and harvested in two different areas of Spain, specifically in Almería (36°50′17.3″ N 2°27.584′ O; Spain) (nLT: non-local tomato) and Tarragona 41°4′29.24″ N 1°3′8.78″ E; Spain) (LT: local tomato), were used. Tomatoes were purchased in autumn, i.e., November 2018, from a local market (Tarragona, Spain) at commercial maturity. Once washed, the edible parts were removed, minced, and frozen in liquid nitrogen. They were then ground, mixed, and lyophilized using the Telstar LyoQuest lyophilizer (Thermo Fisher Scientific, Barcelona, Spain) at −55 °C. The composition of dietary components in dry weight (DW) was determined. LT presented 0.14 g/g DW of proteins; 0.03 g/g DW of total lipids; 0.50 g/g DW of carbohydrates, of which 0.37 mg/g DW were simple sugars; and 0.25 g/g DW of dietary fiber and 0.09 g/g DW of ash. On the other hand, nLT were characterized by having 0.13 g/g DW of proteins; 0.02 g/g DW of lipids; 0.52 g/g DW of carbohydrates, of which 0.40 g/g DW were simple sugars; 0.24 g/g DW of dietary fiber; and 0.09 g/g DW of ash. The profile of phenolic compounds was determined by ultra-high performance liquid chromatography (uHPLC) coupled with mass spectrometry (MS) and previously described by Cruz-Carrión et al. (2022) [18]. Specifically, we determined total (poly)phenols, flavonoids, caffeic and dihydrocaffeic acid derivatives, free phenolic acids, hydroxybenzoic acid derivatives, hydroxycinnamic derivatives, hydroxycinnamoylquinic acids, and phenylpropanoic acid-glycosides. Data are shown in Supplementary Table S1. It should be noted that nLT presented a higher concentration of all phenolic compound types except for total flavonoids.

### 2.2. Determination of Lycopene Content

Lycopene was extracted from the freeze-dried tomato samples using the method described by Motilva et al. (2014) [19]. Samples were weighed (1 g) in test tubes and 5 mL of n-hexane/ethanol/acetone extraction solution (50/25/25, *v*/*v*/*v*) was added to solubilize carotenoids. It was vortexed and centrifuged at 9072× *g* for 15 min. To completely extract the lycopene from the matrix, we proceeded with a second vortex for 15 min and an ultrasound bath for 30 min. The supernatants were decanted with a funnel and 5 mL of water were added. After phase separation, the organic and aqueous phase were collected. With the organic phase the extraction was repeated once more. In the aqueous phase, 5 mL of n-hexane were added, and the extraction was repeated. Finally, the organized phases were pooled, the volume was noted, and the samples were filtered on a 12:22 micron PVDF filter. The resulting solution (105 mL) was injected into an UPLC-DAD chromatographic system using a YMC carotenoid column (4.6 × 150 mm, 3 µM) (Waters Corp., Milford, MA, USA). A two-phase gradient system of methanol (mobile phase A) and methyl tert-butyl ether (MTBE) (mobile phase B) was used. The gradient started in 55% of mobile phase B, reaching 85% at 6 min. The gradient reached initial conditions at 18 min and was kept there in isocratic elution for a further 3 min. Elution was carried out at a flow rate of 0.5 mL/min, and the injection volume was 105 µL lycopene (All-trans-lycopene, Extrasynthese) was used as a standard to detect and compare peaks in tomato samples. The standard was dissolved in n-hexane, and the calibration curve was calculated. Different dilutions within the linearity interval of 0.01–10 mg/L were prepared to achieve the calibration curve. For the detection of lycopene, the chromatograms were recorded at 470 nm. Lycopene was identified based on the retention time of the standard pure compound. The detection and quantification limits can be found in the article by Motilva et al. [19]. It should be noted that we have only quantified all-*trans*-lycopene since it is the predominant carotenoid in tomatoes. Other carotenoid compounds were not considered.

### 2.3. Animals

The guidelines for the care and use of animals in the laboratory were followed, and all the procedures used were evaluated and approved by the Animal Ethics Committee of the Rovira i Virgili University (Tarragona, Spain) (Project identification code: 9495; file number: FUE-2017-00499873).

Seventy-two 8-week-old male Fischer 344 (F344) rats were used for the present experiment. They were placed in cages and in pairs for 4 days at 22 °C and light/dark cycles for acclimatization. After conditioning, they were randomly distributed in one of the three photoperiods: short photoperiod (*n* = 24, L6, 6 h of light and 18 h of darkness); standard photoperiod (*n* = 24, L12, 12 h of light and 12 h of darkness); and long photoperiod (*n* = 24, L18, 18 h of light and 6 of darkness). The amount of light hours for each photoperiod was established in order to simulate the days of the different seasons of the year. Thus, L18 would simulate the summer days, L12 the autumn/spring days, and L6 the winter days. The animals were fed a standard diet (STD) (AO4, Panlab, Barcelona, Spain) and water ad libitum. After 4 weeks, the animals of each photoperiod were randomly distributed in one of the three groups according to the treatment given: LT (*n* = 8), nLT (*n* = 8), or vehicle (VH, *n* = 8). The treatment was carried out for 7 weeks. The supplemented VH was composed of a mixture of sugars that contained the average carbohydrate content of tomatoes used (glucose:fructose 1:1). Regarding the groups supplemented with freeze-dried tomatoes, a dose of 100 mg per body weight diluted in water was daily supplied. The three treatments were administered through a syringe by voluntary licking, guaranteeing the complete taking of the dose. At the end of the experiment, the animals were sacrificed by decapitation, having their last dose of treatment one hour before their death. No anesthetic was administered due to possible interference with the markers studied. The liver and serum obtained were stored at −80 °C.

### 2.4. Serum Analysis

Blood glucose and lipid levels were quantified by enzymatic colorimetric assays: triacylglycerides (TAG), total cholesterol (TC), high-density cholesterol (HDL-c), low-density cholesterol (LDL-c) (QCA, Amposta, Tarragona, Spain), and non-esterified fatty acids (NEFAs) (WAKO, Neuss, Germany). Circulating insulin levels were also quantified using the rat Insulin ELISA kit (Milipore, Barcelona, Spain).

### 2.5. Cardiovascular Risk Indices and HOMA Index

Using the values of the aforementioned biochemical parameters, the following cardiovascular risk markers were calculated: atherogenic index (AI: [Log (TAG/HDL-c)], cardiovascular risk 1 (CR1: TC/HDL-c), cardiovascular risk 2 (CR2: LDL-c/HDL-c), atherogenic coefficient (At.Coef.: [TC-HDL-c]/HDL-c). Values expressed in mmol/L were used for all determinations.

For the calculation of the HOMA index, the blood glucose and insulin values were used in the following equation: (Blood glucose × Insulinemia)/22.5.

### 2.6. Hepatic Gene Expression Analysis

For the extraction of total liver RNA, TRIzol Reagent (Thermo Fisher Scientific, Illkirch-Graffenstaden, France) was used, and the guidelines indicated by the supplier were followed. The cDNA was synthesized by reverse transcription using High-Capacity cDNA Reverse Transcription (Thermo Fisher Scientific, Illkirch-Graffenstaden, France). The specific amplification of the cDNA was carried out thanks to the polymerase chain action in real time (RT-qPCR), using iTaq Universal SYBR Green Supermix (Bio-Rad, Barcelona, Spain). Gene expression analysis was performed using primers obtained from Biomers.net (Ulm, Germany). Supplemental Table S2 shows the corresponding sequences. The mRNA concentration of genes related to liver lipid metabolism was studied: carnitine palmitoyltransferase 1-α (Cpt1α), acetyl-coenzyme A carboxylase (Acc1), fatty acid translocase homolog of CD36 (Cd36), fatty acid synthase (Fas1), sterol regulatory element-binding protein 1 (Srebp-1c), hydroxyacyl-CoA dehydrogenase (Had), and fatty acid transporter 5 (Fatp5). L12-VH group was used as a control group to calculate the relative gene expression, considering that the most characteristic season of the Ektasis tomato is autumn and therefore the standard days. Taking into consideration the efficiency of each primers, the method proposed by Pfaffl [20] was used, and the Ppia gene was used as housekeeping.

### 2.7. Statistical Analysis

The results were expressed as mean ± SEM. SPSS Statistics 22 software (SPSS Inc., Chicago, IL, USA) was used to rule out outliers and for statistical analysis. Normality and homogeneity was evaluated using the Shapiro–Wilk test and Levene’s test, respectively. A two-way analysis of variance (two-way ANOVA) was performed to analyze the effect between the two main factors: photoperiod and treatment. A one-way analysis of variance (one-way ANOVA) was used for those cases in which the two-way ANOVA analysis was significant. In this instance, the following groupings between photoperiods and treatments were made, namely L6-nLT, L6-LT, L6-VH, L12-nLT, L12-LT, L12-VH, L18-nLT, L18-LT, and L18-VH, and we analyzed the differences between means by post hoc test using DMS. For the comparison analysis between means within the same photoperiod or comparing the same treatment in different photoperiods, a Student’s *t*-test was also performed. In cases where the requirement of normality or homogeneity was not met, the non-parametric Kruskal–Wallis and Mann–Whitney U tests were performed as appropriate. The threshold of statistical significance was established at *p* < 0.05 and the trend in 0.05 < *p* < 0.1.

## 3. Results

### 3.1. Lycopene Content

The all-trans-lycopene content (mg/100 g) in freeze-dried samples was 0.72 ± 0.03 and 1.45 ± 0.018 in LT and nLT, respectively, being significantly higher in nLT (*p* = 0.050). Specifically, more than twice the content of this compound was found with respect to LT.

### 3.2. Biochemical Parameters

The results of the biochemical parameters are shown in Table 1. The treatments affected circulating TAG levels in different ways (T, *p* = 0.02, two-way ANOVA), mainly in animals exposed to short days, i.e., L6. In this sense, those that received LT showed a lower concentration of TAG than those that received nLT or VH (*p* = 0.046; *p* = 0.000, respectively). On the contrary, the L6-nLT animals showed statistically higher amounts with respect to their VH (*p* = 0.050). Furthermore, this group presented a higher concentration of TAG than those who received nLT but in different photoperiods, this difference being significant only with those exposed to L18 (*p* = 0.023; *p* = 0.055; L6-nLT vs. L18-nLT; L6-nLT vs. L12-nLT, respectively) (Table 1).

On the other hand, the photoperiod tended to affect the concentration of serum NEFAs (P, *p* = 0.085, two-way ANOVA). Specifically, the effect was observed among the animals that were exposed to either L6 or L18. In this sense, the L6-nLT group showed the highest level compared to the L18-nLT group (*p* = 0.043), whilst no difference was observed between their respective vehicles. Similarly, this difference was also maintained when comparing the animals exposed to L12 although it did not reach statistical significance (*p* = 0.092) (Table 1).

Although an effect of the photoperiod, treatment, or of the interaction of both variables was not observed, there were differences in the concentration of TC between the different experimental groups (Table 1). Specifically, the animals treated with LT in L18 showed the lowest levels of TC in relation to both those that received another treatment in the same photoperiod and to the animals supplemented with LT in the other photoperiods. However, this difference was only statistically significant with their respective VH and the L12-LT group. (*p* = 0.029; *p* = 0.081; *p* = 0.020, *p* = 0.052 L18- LT vs. L18-VH; L18-LT vs. L18-nLT; L18-LT vs. L12-LT; L18-LT vs. L6-LT, respectively) (Table 1). It should be noted that tomato consumption, regardless of the origin, in L12 and L6 seems to increase the TC level in relation to their respective VH. On the other hand, in L18, this response was totally contrary even though these differences did not reach statistical significance.

On the other hand, neither HDL-c nor LDL-c levels were significantly affected by the variables when performing a two-way ANOVA analysis. However, it can be observed in Table 1 that the intake of tomato in L18, regardless of the origin, increases the concentration of HDL-c, this effect being significant with nLT (L18-nLT vs. L18-VH *p* = 0.035, L18-LT vs. L18-VH *p* = 0.174, Student’s *t*-test). Furthermore, LDL-c levels were also lower in animals that received LT or nLT at L18 although this difference did not reach statistical significance. It is important to highlight that a photoperiod effect has been observed among the VH of the groups exposed to L18 and L6. Specifically, the L18-VH animals tended to present the lowest levels of HDL-c in comparison with those L6-VH (*p* = 0.092, Student’s *t*-test).

The two-way ANOVA analysis demonstrated a differential effect on glucose concentration, both in the type of photoperiod and in the type of treatment to which the animals were exposed (P, *p* = 0.003; T, *p* = 0.049). In this sense, the animals exposed to L12 showed higher levels of glucose than those exposed to the other photoperiods, with this effect being significant between L12-VH and L18-VH (*p* = 0.023; *p* = 0.073; L12-VH vs. L18-VH; L12-VH vs. L6-VH, respectively, one-way ANOVA). Moreover, the L6-LT animals presented the lowest glucose levels compared to the others treated within the same photoperiod and even with those that consumed these fruits and were exposed to L12 (L6-LT vs. L6-VH *p* = 0.024, L6-LT vs. L6- nLT *p* = 0.027, L6-LT vs. L12-LT *p* = 0.005) (Table 1).

Insulinemia levels were mainly affected by exposure to different photoperiods (P, *p* = 0.045, two-way ANOVA) (Table 1). In general, the groups exposed to L6 had lower concentrations than those exposed to L12. Specifically, the animals treated with VH showed lower insulinemia than those exposed to L12 (*p* = 0.017 one-way ANOVA). Similarly, the L6-VH group also tended to have a lower insulin concentration than that of L18-VH (*p* = 0.081).

On the other hand, the photoperiod significantly affected the levels of the HOMA index between the experimental groups (P, *p* = 0.008, one-way ANOVA) (Table 1). Specifically, it has been observed that the L12-VH animals presented a statistically higher index compared to those that received the same vehicle, but in the other photoperiods, this difference was statistically significant with those exposed to L6 (*p* = 0.004; *p* = 0.063; L12-VH vs. L6-VH; L12-VH vs. L18-VH, respectively)). Furthermore, the L6-VH group also tended to present a lower HOMA index than those exposed to L18 (*p* = 0.060, Student’s *t*-test)

Exposure to different photoperiods and supplementation with tomatoes from different origins jointly exerted a significant effect on CR1 and At.Coef. (PxT, *p* = 0.007, two-way ANOVA) (Table 1). On the one hand, the consumption of LT in season (L12), tended to decrease the CR1 and the At.Coef. in comparison with its VH (*p* = 0.087, one-way ANOVA; *p* = 0.031, Student’s *t*-test). Furthermore, this L12-LT group also showed a decrease in these parameters with respect to those that consumed LT in L6 (*p* = 0.023). Moreover, tomato consumption, regardless of origin, decreased the CR1 and At.Coef. of the animals exposed to L18, this difference being statistically significant between those that received nLT and a trend among those that received LT (CR1: *p* = 0.029; *p* = 0.075; At.Coef.: *p* = 0.023; *p* = 0.063, respectively). On the other hand, among the animals treated with VH, the exposure to short days (L6) significantly decreased both the CR1 and the At.Coef. in comparison with the exposure to the rest of the photoperiods.

On the other hand, neither AI nor CR2 were significantly affected by exposure to different photoperiods or supplementation with different treatments. However, some differences were observed between the groups. Specifically, the animals that received tomato at L12 or L18 showed a reduction in AI when compared with their respective VH. On the contrary, the LT consumption out of season, in L6, increased this parameter. However, the differences did not reach statistical significance possibly due to the great variability that exists.

Interestingly, with regard to CR2, the L12-VH group showed a significantly lower risk than those animals treated with any of the two tomatoes under evaluation (L12-VH vs. L12-nLT *p* = 0.037, L12-VH vs. L12-LT *p* = 0.082, Student’s *t*-test). Similarly, groups exposed to short days and treated with nLT or LT showed higher CR2 than their respective VH. On the contrary, in the L18 animals, tomato consumption, regardless of origin, presented a lower CR2 when compared with their respective VH. However, these differences did not reach statistical significance.

### 3.3. Gene Expression of Enzymes Related to Lipid Metabolism

**Figure 1:**
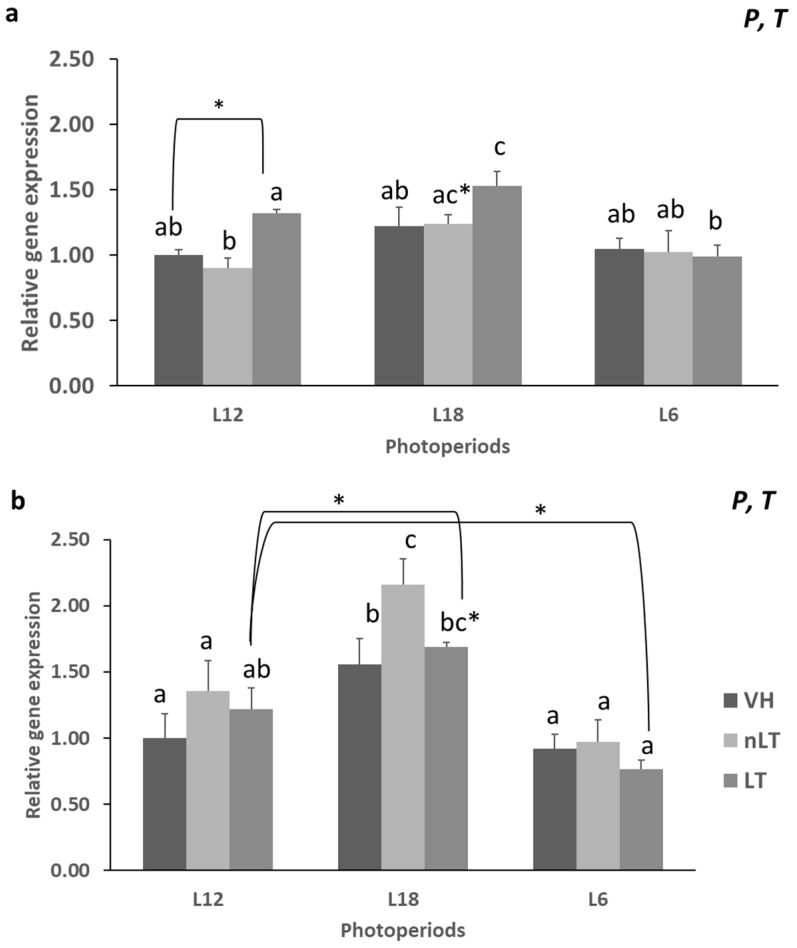
The mRNA levels of acetyl-coenzyme A carboxylase (*Acc1*) (**a**) and fatty acid synthase (*Fas1*) (**b**) of male Fischer 344 rats treated for 7 weeks with vehicle (VH), non-Local tomatoes (nLT) or Local (LT), and exposed to different photoperiods (short: L6, standard: L12 or long: L18). Values expressed as mean ± SEM (*n* = 8). The values were normalized by the L12-VH group. P, photoperiod effect; T, treatment effect (two-way ANOVA, *p* < 0.05). Different letters above the bars indicate significant differences (*p* < 0.05) (post hoc DMS, one-way ANOVA). * Indicate trend (0.05 < *p* < 0.1).

Both the exposure to different photoperiods and the consumption of different treatments had a significant effect on the gene expression of Acc1 and *Fas1* (P, *p* = 0.001, *p* = 0.00; T, *p* = 0.028; *p* = 0.038, respectively). As it can be seen in Figure 1a, the consumption of LT in its consumption season, L12, produced a significant increase in the gene expression of the enzyme *Acc1* with respect to the ingestion of nLT (*p* = 0.014), while it tended to increase it with respect to those animals that received VH (*p* = 0.056). Similarly, consumption of LT in L18 also produced this increase in mRNA concentration with respect to its respective VH (*p* = 0.032) but did not reach statistical significance compared to the group that received nLT (*p* = 0.052). Furthermore, exposure to different photoperiods had a noticeable effect when observing animals that received LT. In this sense, the animals that ingested LT in L12 or L18 showed a statistically higher gene expression compared to those that received the same treatment but in L6 (*p* = 0.034; *p* = 0.00, respectively).

On the other hand, with regard to the gene expression of *Fas1*, a significant effect of the photoperiod was observed in L18 since L18-VH, L18-nLT, as well as L18-LT group presented a higher concentration of hepatic *Fas1* mRNA than the animals that received the same treatments but in the L6 or L12 photoperiods. Furthermore, a differential effect of the treatment was observed in those animals that ingested nLT since in all photoperiods they presented higher gene expression of *Fas1* than the rest of the treatments, this difference was statistically significant only in the animals exposed to long days (*p* = 0.012, *p* = 0.055; L18-nLT vs. L18-VH, L18-nLT vs. L18-LT, respectively). It should be noted that in all photoperiods, the animals that received tomato, regardless of their origin, had a higher *Fas1* gene expression than their respective VH. Except for the L6-LT group, which was the only one that had a lower expression. However, in most cases, they did not reach statistical significance (Figure 1b). 

**Figure 2:**
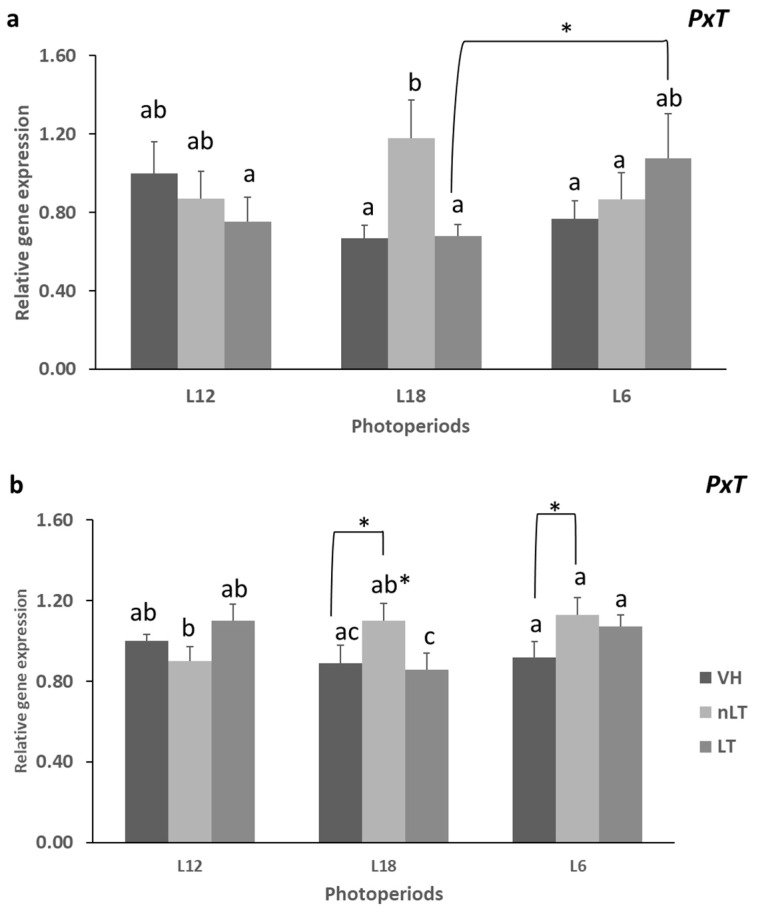
The mRNA levels of translocase homolog of CD36 (*Cd36*) (**a**) and hydroxyacyl-CoA dehydrogenase (*Had*) (**b**) of male Fischer 344 rats treated for 7 weeks with vehicle (VH), non-local tomatoes (nLT), or local (LT) and exposed to different photoperiods (short: L6, standard: L12, or long: L18). Values expressed as mean ± SEM (*n* = 8). The values were normalized by the L12-VH group. PxT, effect of interaction (two-way ANOVA, *p* < 0.05). Different letters above the bars indicate significant differences (*p* < 0.05) (post hoc DMS, one-way ANOVA). * Indicate trend (0.05 < *p* < 0.1).

The interaction between photoperiod and treatment exerted a statistical effect on the levels of gene expression of the enzymes *Cd36* and *Had* (PxT: *p* = 0.030; *p* = 0.032, two-way ANOVA, respectively). As it can be seen in Figure 2a, the *Cd36* mRNA concentration was statistically higher in those animals that consumed nLT in L18, with respect to those that were exposed to the same photoperiod but received the other treatments (*p* = 0.020, *p* = 0.018, L18-nLT vs. L18-VH; L18-nLT vs. L18-LT, respectively). However, in the rest of the groups exposed to L6 or L12, this differential effect was not observed between the treatments. Furthermore, the L18-nLT animals also showed higher Cd36 gene expression compared to those L6-nLT (*p* = 0.044). However, on the contrary, the L18-LT group tended to present a lower concentration of mRNA of the enzyme with respect to the L6-LT (*p* = 0.060).

Similarly, it can be observed in Figure 2b that the L18-nLT animals also showed a higher gene expression of the *Had* enzyme compared to those that received LT in the same photoperiod (*p* = 0.026). Furthermore, both the VH exposed to L18 and those exposed to L6 tended to present a lower concentration of mRNA than the animals treated with nLT in their respective photoperiods (*p* = 0.053, *p* = 0.053, respectively). However, those exposed to L12 this relationship was the opposite although it did not reach statistical significance. Further, in relation to the groups that received LT, those exposed to L18 were the ones that showed a significantly lower expression with respect to the other photoperiods (*p* = 0.026; *p*= 0.050; L12, LL6, respectively). On the other hand, in relation to those supplemented with nLT, it was those exposed to L12 that showed a lower concentration of *Had* mRNA compared to L6 or L18.

**Figure 3:**
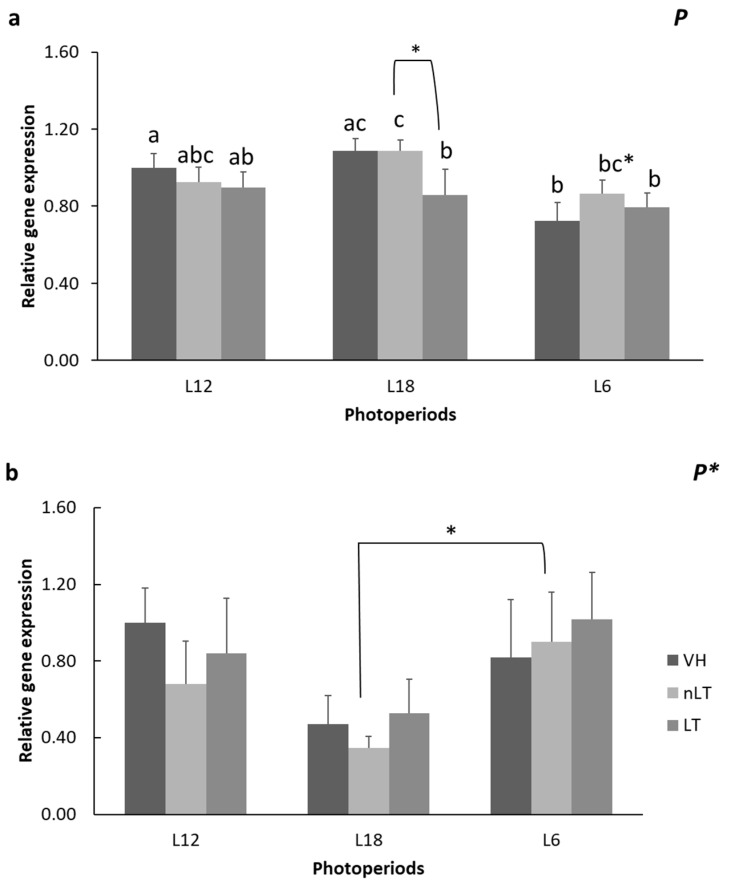
The mRNA levels of sterol regulatory element-binding protein 1 (Srebp-1c) (**a**) and carnitine palmitoyltransferase 1-α (*Cpt1α*) (**b**) of male Fischer 344 rats treated for 7 weeks with vehicle (VH), non-local tomatoes (nLT), or local (LT), and exposed to different photoperiods (short: L6, standard: L12, or long: L18). Values expressed as mean ± SEM (*n* = 8). The values were normalized by the L12-VH group. P, photoperiod effect (two-way ANOVA, *p* < 0.05). Different letters above the bars indicate significant differences (*p* < 0.05) (post hoc DMS, one-way ANOVA). * Indicate trend (0.05 < *p* < 0.1).

The gene expression of the Srebp-1c protein was significantly affected by exposure to different photoperiods, while the expression of *Cpt1α* tended to be affected by the same variable (P, *p* = 0.005; *p* = 0.083, two-way ANOVA, respectively). In this sense, as it can be seen in Figure 3a, the L6-VH animals were those that presented a lower concentration of mRNA, with respect to the other VH (*p* = 0.026, *p* = 0.001; L6-VH vs. L12-VH, L6-VH vs. L18-VH, respectively). Similarly, the L6-nLT group tended to present a lower expression of Srebp-1c than those exposed to L18 (*p* = 0.063). On the other hand, among the animals exposed to long days, L18, those that received LT had a greater amount of mRNA compared to the rest of the treatments, reaching statistical significance only with the group treated with VH (*p* = 0.022, *p* = 0.061; L18-LT vs. L18-VH, L18-LT vs. L18-nLT, respectively). No significant differences have been observed between the treatments of the other photoperiods.

Additionally, the photoperiod influenced the gene expression of *Cpt1α* since it was observed that the animals that received nLT in L6 tended to present a higher concentration of mRNA of the enzyme compared to those that received the same treatment but were exposed to L18 (*p* = 0.077) (Figure 3b).

**Figure 4:**
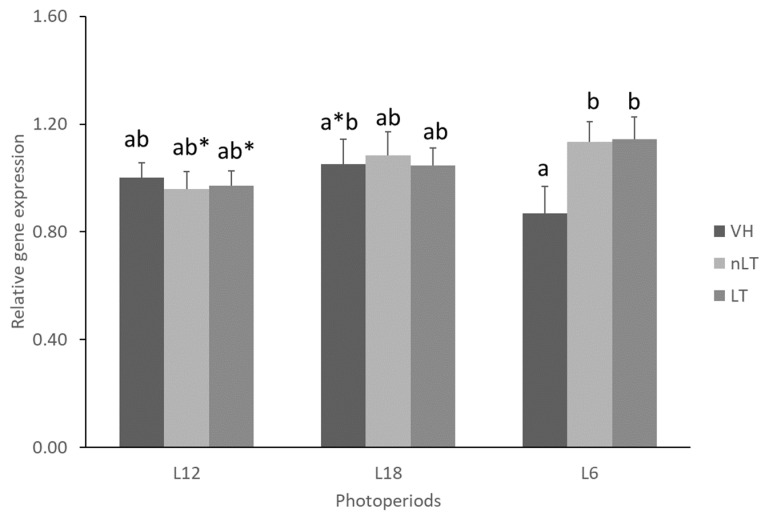
The mRNA levels of fatty acid transporter 5 (Fatp5) of male Fischer 344 rats treated for 7 weeks with vehicle (VH), non-local tomatoes (nLT), or local (LT) and exposed to different photoperiods (short: L6, standard: L12, or long: L18). Values expressed as mean ± SEM (*n* = 8). The values were normalized by the L12-VH group. Different letters above the bars indicate significant differences (*p* < 0.05) (post hoc DMS, one-way ANOVA). * Indicate trend (0.05 < *p* < 0.1).

Although neither the photoperiod nor the treatment significantly affected the *Fatp5* gene expression, it can be seen that there were differences between the treated animals. Specifically, those who received tomato, regardless the origin, in L6, presented a higher concentration of mRNA of the *Fatp5* transporter than their respective VH (*p* = 0.014, *p* = 0.010; L6-VH vs. L6-nLT, L6-VH vs. L6-LT, respectively). Similarly, they also tended to show higher gene expression than the groups treated but exposed to L12. Furthermore, the L18-VH group tended to present a higher amount of mRNA than the L6-VH group although it did not reach statistical significance (*p* = 0.094) (Figure 4).

## 4. Discussion

In the present study, it has been evaluated how the origin of the tomato and its time of consumption can influence certain metabolic parameters as well as the gene expression of certain key liver enzymes of lipid metabolism. Moreover, we demonstrate how these differences can be given by the characteristic composition in bioactive compounds that could be determined by the geographical origin of the plant.

First, regarding their content in bioactives, we observed that nLT had a higher content of all-trans-lycopene than LT. Tomato is a climacteric fruit, where ripening is carried out by an increase in oxygen consumption and production of carbon dioxide and ethylene [21]. In fact, the sharp peak in ethylene production is considered one of the most important factors that determine the physical and organoleptic characteristics and the content of bioactive compounds such as polyphenols and lycopene [22]. There are two types of fruit ripening: physiological ripening, which is when development is complete, and commercial ripening, which is required by the market [23]. Considering that the tomato is a climacteric fruit, once pulled from the plant, it continues to breathe and develop, and they are generally harvested in early stages of maturation to avoid reaching the consumer excessively ripe [24]. Consequently, we infer that the higher content of lycopene and total polyphenols in nLT may be because they reached the peak of ethylene and concentration of bioactive compounds at times close to their use. On the other hand, because the LT are nearby, they are harvested in late stages of maturation, where the ethylene peak was recent and therefore begins a stage of decomposition and loss of bioactive compounds. Furthermore, storage conditions also influence the content of bioactive compounds in tomatoes [10]. It has been observed that the amount of polyphenols and lycopene can vary between two and three times between tomatoes of different varieties and origins. [10,12]. Therefore, according to the xenohormesis theory, the metabolic effects and their health implications may also vary according to the place of origin of the ingested fruit thanks to the fact that the phytochemical composition of each one would produce adequate adaptive changes for better survival [14]. A decrease in oxidative stress markers and plasma lipids are some of the observed effects of consuming locally produced fruits [15,16,25]. Even though there are differences in the climatic conditions of the different tomato harvest sites, we identified one limitation in the study as not assessing the climatic and agronomic conditions when tomatoes were harvested. Consequently, we encourage future studies to take these considerations into account.

*The photoperiod affects the lipid and carbohydrate metabolism of all treatments.*

In the present study, we observed that the photoperiod significantly affects the plasma glucose and insulin concentration, the HOMA index, and the hepatic gene expression of *Acc1, Fas1,* and Srebp1c. Furthermore, the photoperiod tends to affect the concentration of plasma NEFAs and the levels of *Cpt1α* mRNA. Specifically, it has been observed that those L6-VH animals presented lower amounts of plasma glucose and insulin and consequently a lower HOMA index than those L12-VH. In addition, they also presented a lower gene expression of Srebp-1c. Therefore, because the animals exposed to L6 had more hours of activity, it is to be expected that they have less blood glucose than those exposed to L12. It is important to note that glucose and insulin stimulate the transcriptional activation of several lipogenic genes through complex mechanisms [26]. In this sense, insulin activates the expression and activation of Srebp-1c through the PI3-kinase/AKt pathway and consequently stimulates de novo lipogenesis [27]. Therefore, the L12-VH group presented a higher glycemia, which could have produced a greater release of insulin and consequently could have stimulated a greater expression of Srebp-1c. However, this higher expression did not result in an induction of lipogenic genes such as *Acc1* and *Fas1* or in a higher concentration of plasma TAG. In this sense, it has been observed that other transcription factors are necessary to regulate the transcription of lipogenic genes. [26].

On the other hand, the effect of the photoperiod has also been observed among animals that received nLT and were exposed to L6 or L18. Specifically, those exposed to L6 showed significantly higher amounts of serum NEFAs and TAG and tended to higher gene expression of *Cpt1α*. Furthermore, a lower concentration of *Fas1* and *Cd36* mRNA has also been observed, together with a trend towards a lower expression of the Srebp-1c protein than the L18-nLT animals. It should be noted that when there is fasting or prolonged periods of exercise, there is hydrolysis of the TAG stored in the different fatty deposits and consequently the plasma NEFAs rise to supply other tissues with energy [27]. Therefore, it is not strange to observe a higher hepatic expression of *Cpt1α* in L6-nLT animals, which limits the passage of fatty acids (FA) to the mitochondria for oxidation [28]. However, sometimes the final fate of NEFAs is not oxidation but also re-esterification to TAG [29]. However, L6-nLT animals tended to have lower hepatic expression of the Srebp-1c protein, which controls the activation of enzymes for the synthesis of TAG and FA, including *Fas1*, whose expression is also diminished. Moreover, these animals presented a lower expression of *Cd36*, which is a transporter of plasma NEFAs into the hepatocytes [27]. Moreover, considering that fewer hours of light is associated with greater activity in rodents [30] and that lipids mobilized from adipose tissue may be elevated up to two hours after exercise [31], elevated TAGs in L6-nLT could be due to increased trafficking from peripheral tissues as energy sources. Therefore, the marked difference between the hours of activity between groups L6 and L18 could be reflected in the concentration of plasmatic energetic substrates. However, when compared with the L12 group, these differences did not reach statistical significance. In this sense, a limitation of the study was the lack of analysis of the gene expression of enzymes and the quantification of markers related to energy expenditure and carbohydrate and lipid metabolism in metabolically active tissues, such as adipose tissue or muscle. For a better understanding of metabolism in different photoperiods as well as what happens with tomato supplementation from different geographical origins, we suggest future research on these tissues. Likewise, a record of activity and food consumption in 24 h in the different experimental groups would be useful to clarify the mechanisms involved. 

Among the animals that received LT, an effect of exposure to different photoperiods was also observed, mainly among those that were exposed to short days. On the one hand, the L6-LT group presented a glycemia and gene expression of Acc1 significantly lower than those exposed to L12, together with a tendency to a lower expression of *Fas1* and a higher expression of *Fatp5*. Importantly, Fatp5 localizes to hepatocyte membranes and mediates uptake of long-chain FA from the circulation into the cell [27]. FATP5-ko animals have been shown to reduce FA uptake by up to 50% [32]. Therefore, it can be assumed that the L6-LT animals presented a greater uptake of hepatic FA through Fatp5, while they expressed less amount of lipogenic enzyme mRNA. Similarly, the L6-LT animals presented a lower concentration of *Acc1* and *Fas1* mRNA and a higher *Had* level, while they showed a tendency to express more *Cd36* than the L18-LT animals, indicating a lower synthesis and higher FA oxidation. However, these results are not consistent with those reported by other authors since there is evidence that exposure to fewer hours of light produces a greater synthesis of circulating TAG due to an activation of lipogenic genes, including Fas1, because of a low level of NR1D2 [26]. However, these studies were carried out with animals fed a standard diet and in the absence of tomato. Consequently, one could think of a particular effect of the treatment, which will be discussed later.

*Tomato consumption has differential effects on lipid metabolism depending on its geographical origin.*

In the present study, a significant treatment effect has been observed between the biochemical parameters and between the gene expression of key enzymes in lipid metabolism. Specifically, a clear effect of the geographical origin of the tomatoes is observed between the animals L6 because the L6-LT had lower levels of TAG and glucose than L6-VH, while the group L6-nLT had higher TAG levels than L6-VH, whereas blood glucose was not significantly affected. In this sense, there are numerous studies where an improvement in the lipid and glycemic profile was observed when supplementing the animals with tomato. However, most of them use high-fat diet models or animals with an established metabolic pathology to observe these differences [33,34]. Unfortunately, there is little bibliography on the preventive effect of its intake in a healthy animal model and studies that investigate the molecular mechanisms of this effect are required. A hypoglycemic and lipid-lowering effect of the intake of polyphenolic compounds has been observed because of their ability to inhibit the activity of digestive enzymes and reduce the hydrolysis of carbohydrates and fats [35]. However, in our study, the decrease in TAG and glucose has not been observed in all the groups that consumed tomato but only in L6-LT. This effect may be due to the higher concentration of flavanols contained in LT compared to nLT observed in our previous publication [18].

On the other hand, an effect of the treatment was also observed on the gene expression of enzymes related to lipid metabolism in animals exposed to L18. Specifically, those who received nLT showed a higher concentration of *Fas1* and *Cd36* mRNA, together with a tendency to an increased *Had* expression in relation to their respective VH. Differences were also observed between the groups that received nLT and LT. Notably, Cd36 is a transmembrane transporter for carotenoids, such as lycopene [36]. Therefore, it is not surprising that the nLT, having presented a greater amount of lycopene than the LT, also had a higher gene expression of its transporter. In this sense, Elvira-Torales et al. (2018) observed a higher expression of lipid transporters, including *Cd36* and *Fatp5* in animals that received tomato, assuming a greater entry of lipids to the hepatocyte [37].

*The consumption of TL in season, L12, decreases CR1 and At.Coef.*

In the present study, we observed the metabolic consequences of the intake of tomatoes from different geographical origins together with the effects of its consumption in different photoperiods (PxT effect), which simulate different seasonal times. It should be noted that the Ekstasis variety used is harvested in the autumn season, which is simulated by the L12 photoperiod. Specifically, despite not having differences in the concentration of serum biochemical parameters, the animals L12-LT presented lower CR1 and At.Coef. than their respective VH. In contrast, L6-LT did not present different serum cholesterol levels but had higher CR1 and At.Coef. than their respective VH. These results would indicate the importance of seasonal consumption and the local origin of the fruit on markers of atherogenic risk. CR1 is a more sensitive and specific cardiovascular risk index than the TC concentration since it is an indicator of the formation of coronary plaques and the thickness of the intima media in the carotid arteries [38]. Furthermore, the At.Coef. is also a good predictor of cardiovascular risk since it reflects the atherogenic potential of non-HDL-c cholesterol, thus including LDL-C, VLDL-c, and IDL-c [39,40]. Similarly, we previously observed that when tomatoes are consumed in their season, they improve or maintain antioxidant biomarkers, reducing oxidative stress compared to their consumption in other seasons [16].

On the other hand, in the animals exposed to L18 and supplemented with tomato, they showed a lower concentration of TC than their respective VH. However, in the other photoperiods, this difference was not observed, showing an overall effect of both the photoperiod of consumption and the origin of the tomato. In this sense, serum cholesterol levels depend on the balance between the consumption of products that contain it and de novo cellular synthesis. The enzymes 3-hydroxy-3-methylglutaryl coenzyme A (HMG-CoA) reductase and cholesterol acyl transferase as well as the cellular receptors for LDL-c play a key role in the regulation of cholesterol metabolism [41]. In agreement with our findings, a hypocholesterolemic effect in animals that consumed tomato juice together with a normal diet was observed, and we attribute this effect to an post-transcriptional inhibition in the activity and of the HMGCR enzyme [42]. Some phenolic compounds, such as flavonoids, have a high affinity with the HMG-CoA enzyme, either by hydrophobic Van de Walls bonds or by the numerous -OH groups, which produce a union of these bioactive compounds, causing inhibition and consequent decrease in cholesterol synthesis [42]. It should be noted that according to our previous publication, LT has a greater amount of total flavanols than nLT, which could explain this decrease. Furthermore, it has been observed that rats supplemented with tomato juice increased in mRNA expression of the liver X receptor, which stimulates the excretion of bile acids by activating CYP7A1 [37]. Therefore, a decrease in the synthesis and a greater excretion would be the possible mechanisms by which the tomato would exert its hypocholesterolemic effect. However, this effect is not observed in the other animals treated in the rest of the photoperiods, so it could be inferred in an overall effect of the treatment and the exposure to L18.

## 5. Conclusions

In conclusion, in this study, we obtained evidence that the metabolic effects of tomato consumption depend not only on the season of consumption but also on the geographical origin of the fruit. Specifically, (**a**) in the tomato consumption season, L12, a beneficial effect of the intake of LT was observed on the cardiovascular risk markers CR1 and At.Coeff.; (**b**) off-season, in L6, caused an opposite effect on these parameters; (**c**) however, L6-LT caused a decrease in blood glucose and TAG; (**d**) on the contrary, consumption of nLT in L6 increased them; (**e**) and in L18, it caused an increase in the gene expression of *Cd36*, *Had*, and *Fas1*. These effects could be due to the higher lycopene content found in nLT. However, the same effect was not observed in all photoperiods. Consequently, more studies on the interaction of these bioactive compounds with other metabolic enzymes are required to understand the variation that exists between the consumption of phytochemicals, the use of energy substrates, and the number of hours of light and darkness per day.

## Figures and Tables

**Table 1:** Plasma parameters and atherogenic indices in Fischer 344 rats exposed to different photoperiods and supplemented with local or non-local tomato lyophilizate or vehicle for 7 weeks.

	*L6*			*L12*			*L18*			*2wA*
	VH	nLT	LT	VH	nLT	LT	VH	nLT	LT	
Serum Parameters										
TAG (mmol/L)	1.14 ± 0.11 c	1.40 ± 0.07 a	0.88 ± 0.09 b	1.17 ± 0.09 c	1.15 ± 0.10 a*c	0.99 ± 0.08 bc	0.95 ± 0.08 bc	1.10 ± 0.10 b*c	0.95 ± 0.08 bc	T
TC (mmol/L)	1.80 ± 0.16 ab	2.03 ± 0.26 a	2.15 ± 0.25 ab*	1.97 ± 0.20 ab*	2.02 ± 0.23 ab*	2.15 ± 0.25 a	2.11 ± 0.24 a	1.96 ± 0.21 ab*	1.39 ± 0.13 b	
HDL-c	0.75 ± 0.12	0.80 ± 0.21	0.64 ± 0.22	0.50 ± 0.12	0.53 ± 0.01	0.66 ± 0.14	0.38 ± 0.16	0.85 ± 0.11	0.67 ± 0.12	
LDL-c	0.16 ± 0.06	0.14 ± 0.06	0.19 ± 0.08	0.09 ± 0.03	0.15 ± 0.04	0.14 ± 0.03	0.13 ± 0.02	0.08 ± 0.03	0.09 ± 0.02	
NEFAs	0.79 ± 0.10 ab	1.04 ± 0.27 a	0.87 ± 0.09 ab	0.78 ± 0.06 b	0.76 ± 0.10 a*b	0.77 ± 0.11 b	0.76 ± 0.08 ab	0.70 ± 0.04 b	0.62 ± 0.06 b	P *
Glucose	8.87 ± 0.53 ac*	8.79 ± 0.55 ac*	7.20 ± 0.27 b	10.18 ± 0.55 c	9.52 ± 0.64 ac	9.22 ± 0.42 ac	8.50 ± 0.19 a	8.71 ± 0.45 a	8.27 ± 0.56 ab	T, P
Insulin (ng/mL)	5.38 ± 0.68 b	6.27 ± 0.86 ab	6.68 ± 1.24 ab	9.93 ± 1.53 a	7.82 ± 1.10 ab	8.05 ± 1.63 ab	8.80 ± 0.54 ab*	6.78 ± 1.30 ab	8.61 ± 1.60 ab*	P
**Ratios**										
HOMA	0.09 ± 0.01 a	0.11 ± 0.02 a	0.09 ± 0.02 a	0.19 ± 0.04 b	0.14 ± 0.02 ab	0.14 ± 0.03 ab	0.13 ± 0.01 ab*	0.11 ± 0.02 a	0.13 ± 0.03 a	P
Atherogenic Index	0.23 ± 0.12	0.21 ± 0.10	0.21 ± 0.10	0.45 ± 0.12	0.29 ± 0.05	0.24 ± 0.13	0.48 ± 0.24	0.14 ± 0.04	0.19 ± 0.09	
CR1	1.97 ± 0.34 b	2.91 ± 0.71 abc*	4.67 ± 1.15 c	3.97 ± 0.51 a	3.34 ±0.53 ab	2.17 ± 0.52 a*b #	4,71 ± 1.59 a	2.09 ± 0.11 b	2.71 ± 0.43 a*b	PxT
CR2	0.14 ± 0.07 a*b	0.24 ± 0.12 ab	0.26 ± 0.07 ab	0.10 ± 0.03 b	0.40 ± 0.12 a	0.34 ± 0.13 ab*	0.33 ±0.13 ab	0.13 ± 0.05 ab	0.26 ± 0.09 ab	
At.Coef	0.97 ± 0.34 b	2.24 ± 0.73 abc*	3.67 ± 1.15 c	2.97 ± 0.51 a	2.34 ± 0.53 ab	1.17 ± 0.52 a*b #	3.71 ± 1.59 a	1.09 ± 0.11 b	1.71 ± 0.43 a*b	PxT

Fischer 344 rats fed a standard diet were exposed for 7 weeks to a short, standard, or long photoperiod, with 6 (L6), 12 (L12), or 18 (L18) hours of light, respectively, and supplemented with vehicle (VH) or with local tomato originating in Tarragona (LT) or non-local originating in Almería (nLT). Data are expressed as the mean ± SEM. (*n* = 8). TAG, triacylglycerides; TC, total cholesterol; HDL-c, high-density lipoprotein cholesterol; LDL-c, low-density lipoprotein cholesterol; NEFAs, non-esterified fatty acids; HOMA, homeostasis model assessment; CR1 and CR2, cardiovascular risk 1 and 2, respectively; At.Coef., atherogenic coefficient. Two-way ANOVA analysis (2 × 2 photoperiod factorial design (L6, L12, or L18) × treatment (VH, nLT or LT), was used to assess the differences between groups. P, photoperiod; T, treatment effect; PxT, effect of interaction. Different letters above the bars indicates significant differences (*p* < 0.05) (post hoc DMS, one-way ANOVA). * Indicates trend (0.05 < *p* < 0.1); # indicates significative difference with their respective VH (*p* < 0.05 Student’s *t*-test).

## Data Availability

Not applicable.

## Supplementary Materials

The following supporting information can be downloaded at: https://www.mdpi.com/article/10.3390/nu15010043/s1, Table S1: Concentration of (poly)phenolic compounds in local (LT) and non-local (nLT) Ekstasis tomatoes; Table S2: Nucleotide sequences of primers used for real time quantitative PCR.
