# Supplementary material for: In-Season Consumption of Locally Produced Tomatoes Decreases Cardiovascular Risk Indices
Source: Nutrients. 2022 Dec 22;15(1):43. doi: 10.3390/nu15010043 (PMC9823597; doi:10.3390/nu15010043)
Supplement: Supplementary file 1 [file nutrients-15-00043-s001.zip › nutrients-2066757-supplementary.pdf]

Supplementary Material

Table S1. *Concentration of (poly)phenolic compounds in local (LT) and non-local (nLT) Ekstasis tomatoes.*

Compound	**LT**	**nLT**
Flavonoids	399.32	283.37
Caffeic and dihydrocaffeic acid derivatives	185.34	214.49
Free phenolic acids	71.90	120.22
Hydroxybenzoic acid derivatives	102.34	89.42
Hydroxycinnamic acid derivatives	867.26	881.67
Hydroxycinnamoylquinic acids	1149.83	1560.35
Phenylpropanoic acid-glycosides	586.30	596.02
(Poly)phenolic compounds	3362.30	3745.54

The results are expressed as μg/g dw. Table was adapted from Cruz-Carrión et al. (2002).

Table S2. *Nucleotide sequences of primers used for real time quantitative PCR*.

GENE	***Forward primer (5′ to 3′)***	***Reverse primer (5′ to 3′)***
Acc1	TGCAGGTATCCCCACTCTTC	TTCTGATTCCCTTCCCTCCT
Cd36	GTCCTGGCTGTGTTTGGA	GCTCAAAGATGGCTCCATTG
Cpt1α	GCTCGCACATTACAAGGACAT	TGGACACCACATAGAGGCAG
Fas1	CTATTGTGGACGGAGGTATC	TGCTGTAGCCCAGAAGAG
Fatp5	CCTGCCAAGCTTCGTGCTAAT	GCTCATGTGATAGGATGGCTGG
Had	ATCGTGAACCGTCTCTTGGT	AGGACTGGGCTGAAATAAGG
Srebp-1c	CCCACCCCCTTACACACC	GCCTGCGGTCTTCATTGT

The table shows the nucleotide sequences of primers used for PCR amplification. *Acc1*, acetyl CoA carboxylase; *Cd36*, fatty acid translocase, homologue of CD36; Cpt1*α*, carnitine palmitoyltransferase 1 alpha; Fas1, sterol regulatory element-binding protein 1; *Fatp5*, fatty acid transport protein 5; *Had*, hydroxyacyl-CoA dehydrogenase; *Srebp1c*, sterol regulatory element-binding protein 1c.
